# A Phenolic Acid Decarboxylase-Based All-Enzyme Hydrogel for Flow Reactor Technology

**DOI:** 10.3390/mi10120795

**Published:** 2019-11-20

**Authors:** Esther Mittmann, Sabrina Gallus, Patrick Bitterwolf, Claude Oelschlaeger, Norbert Willenbacher, Christof M. Niemeyer, Kersten S. Rabe

**Affiliations:** 1 Institute for Biological Interfaces (IBG-1), Karlsruhe Institute of Technology (KIT), 76187 Karlsruhe, Germany; esther.mittmann@kit.edu (E.M.); sabrina.gallus@kit.edu (S.G.); patrick.bitterwolf@kit.edu (P.B.); niemeyer@kit.edu (C.M.N.); 2 Institute for Mechanical Process Engineering and Mechanics (MVM), Karlsruhe Institute of Technology (KIT), 76187 Karlsruhe, Germany; claude.oelschlaeger@kit.edu (C.O.); norbert.willenbacher@kit.edu (N.W.)

**Keywords:** biocatalysis, phenolic acid decarboxylase, hydrogel, enzyme immobilization, SpyCatcher/SpyTag, microreactor

## Abstract

Carrier-free enzyme immobilization techniques are an important development in the field of efficient and streamlined continuous synthetic processes using microreactors. Here, the use of monolithic, self-assembling all-enzyme hydrogels is expanded to phenolic acid decarboxylases. This provides access to the continuous flow production of *p*-hydroxystyrene from *p*-coumaric acid for more than 10 h with conversions ≥98% and space time yields of 57.7 g·(d·L)^−1^. Furthermore, modulation of the degree of crosslinking in the hydrogels resulted in a defined variation of the rheological behavior in terms of elasticity and mesh size of the corresponding materials. This work is addressing the demand of sustainable strategies for defunctionalization of renewable feedstocks.

## 1. Introduction

The current transformation of the chemical industry calls for a constant intensification of the use of biomass as feedstock for the sustainable synthesis of fine chemicals. However, the high complexity of this renewable raw material and especially the high mass fraction of oxygen in comparison to fossil carbon resources is limiting its widespread utilization. Decarboxylation is an important method for defunctionalization, which can be employed to alleviate these impediments [1,2,3,4,5].

In order to add value to ubiquitously available bio-derived phenolic acids [6,7,8,9,10,11], and to provide alternatives to chemical methods often relying on transition metal catalysis [2,10,12], phenolic acid decarboxylases (PADs) offer an elegant enzymatic route to catalyse the elimination of carbon dioxide from hydroxyphenacrylic acids [13,14,15]. The resulting styrene derivatives are of great industrial relevance since the terminal alkene provides a versatile handle for further chemical functionalization or polymerization reactions [16,17,18]. In contrast to other (de)carboxylases, several PADs found in bacteria [19,20,21,22] and plants [23] act on an acid-base mechanism via a quinone methide intermediate [24] and do not require a cofactor, therefore eliminating the need of a laborious and expensive cofactor regeneration system [25,26].

The versatility of PADs has been demonstrated by integration of the decarboxylation reaction into biocatalytic [6,27] and chemical [28,29,30] reaction cascades. However, limitations can arise due to the product inhibition [28,29] frequently observed for this class of enzymes. The transfer of the process from batch stirred tank reactors to fluidic, micro- and macrostructured reactors can provide a viable solution, as the convective product removal can avoid the accumulation of high and adverse product concentrations [31]. Therefore, much effort is currently devoted to the development of continuous flow reactors employing immobilized enzymes [32,33,34,35,36]. In general, such reactors can be prepared by anchoring the enzyme of interest on the reactor surface using a variety of chemical methods or else by entrapment or crosslinking of the enzymes [34,37,38]. Immobilizing the enzymes on the planar surfaces of a reactor wall can result in a single monolayer, limiting the catalytic performance. Here, employing immobilization strategies which result in three-dimensional structures, can lead to a significant increase of the enzymatic activity per reactor volume. This approach can be facilitated with so-called crosslinked enzyme aggregates or crystals (CLEAs or CLECs) [39,40,41,42,43]. Another advantage of this strategy is the minimization of carrier materials, so that only a small volume of the valuable reactor space is needed for supporting structures. However, since the crosslinking process usually relies on non-specific chemical crosslinkers, this method can lead to the inactivation of enzymes. 

**Figure 1:**
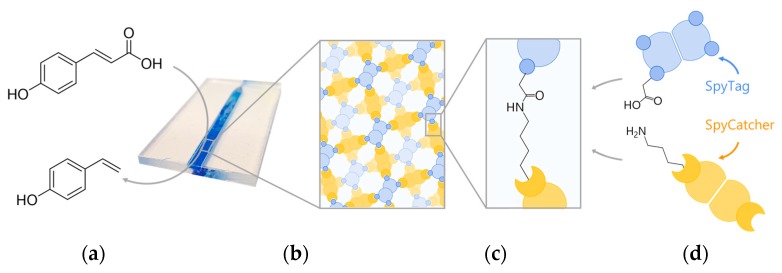
(**a**) Continuous synthesis of substituted styrenes from readily available bio-derived phenolic acids through biocatalytic decarboxylation, as exemplified here for p-hydroxystyrene, is achieved by implementation of a microreactor (**b**). This reactor is filled with an all-enzyme hydrogel (**c**) comprised of the dimeric Phenolic Acid Decarboxylase (PAD), crosslinked via isopeptide bonds spontaneously generated through genetically fused SpyTag/SpyCatcher complexes (**d**). Note that the protein hydrogel loaded into the micro reactor (**b**) is stained with coomassie brilliant blue.

We have recently demonstrated the construction of self-assembling all-enzyme hydrogels constructed from ketoreductases and glucose dehydrogenases, which were genetically fused with either the SpyTag (ST) peptide or the SpyCatcher (SC) protein, respectively [44,45,46]. This highly efficient autocatalytic bioconjugation system is based on the rapid formation of a covalent isopeptide bond through the SpyTag-SpyCatcher complex that occurs under physiological conditions [47,48,49,50]. To render the all-enzyme hydrogel immobilization strategy applicable to a broader range of biocatalysts, we here report, for the first time, on the use of recombinant variants of a highly active homodimeric PAD (molecular weight of a monomer 20 kDa) obtained from *Enterobacter* sp. [51]. By creating SC- and ST-tagged PAD variants we demonstrate the fabrication of all-enzyme hydrogels. The functionality of the novel biocatalytic materials is demonstrated by the continuous production of p-hydroxystyrene in a microfluidic flow reactor (Figure 1). 

## 2. Materials and Methods

All chemicals were purchased from Sigma Aldrich (St. Louis, MI, USA) or VWR (Radnor, PA, USA) if not stated otherwise. 

### 2.1. Synthesis of p-Hydroxystyrene Via Wittig Reaction

**Scheme 1:**
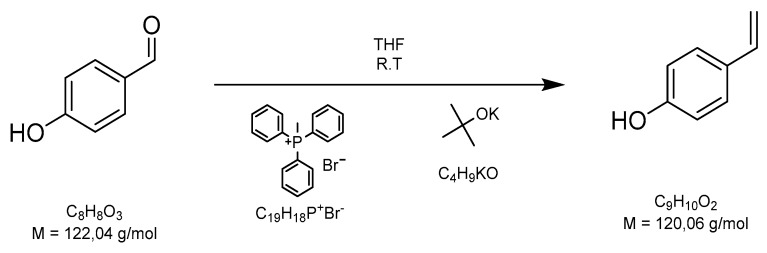
Synthesis of *p*-hydroxystyrene.

For calibration curves and as positive control, *p*-hydroxystyrene was synthesized as described previously (Scheme 1) [52]. In brief, potassium tert-butoxide (≥98%, 2.81 g; 25 mmol) was added to a mixture of methyl triphenylphosphonium bromide (≥98%, 5.36 g; 15 mmol) in anhydrous tetrahydrofurane THF (ACS grade, 20 mL) under argon atmosphere. The mixture was stirred for 10 min at ambient temperature. Subsequently *p*-hydroxybenzaldehyde (≥98%, 1.52 g; 10 mmol) dissolved in THF (10 mL) was added dropwise. The reaction was stirred for 4 h and the product formation was monitored by thin layer chromatography (TLC). After quenching of the reaction mixture with saturated NH_4_Cl solution and the removal of THF under reduced pressure, the crude product was extracted with CH_2_Cl_2_ (analytical grade). The organic layer was washed with a saturated NaCl solution, dried over anhydrous Na_2_SO_4_, and after filtration and evaporation of the solvent, purified by chromatography column (SiO_2_, 5% ethyl acetate in *n*-hexane) to give pure *p*-hydroxystyrene, 834.5 mg (6.945 mmol) in a 69.5% yield as white crystals. R_f_ = 0.44 (16% ethyl acetate in *n*-hexane). The nuclear magnetic resonance (NMR) spectra matched with literature data.

### 2.2. Plasmid Construction

Plasmid construction was carried out using the isothermal recombination as described by Gibson et al. utilizing oligonucleotide primers with 20–30 bp homologous overlaps [53]. The products of the in vitro recombination were then incubated for 1 h at 37 °C with DpnI (New England Bioloabs, Beverly, MA, USA) to ensure the sample did not contain any methylated DNA templates from previous polymerase chain reactions. The purification of dissolved plasmids was carried out using the ZR Plasmid Miniprep-Classic (Zymo Research, Freiburg, Germany). Sequences were verified by commercial sequencing (LGC Genomics, Berlin, Germany). The sequences of the primers can be found in Table 1. pET_PAD-His was used as a template for further cloning steps [30].

pET_PAD-SC-His: The backbone encoding for a N-terminal PAD and C-terminal 6× His-Tag separated by a glycine spacer was amplified using primers EM01 and EM02 with pET_PAD-His as the template. This backbone was then recombined with a SC encoding insert, which had been generated by polymerase chain reaction (PCR) using the primers EM03 and EM04 with pTF16_Lpp-OmpA-SC as the template [48]. pET_PAD-ST-His: The C-terminal ST sequence were inserted into plasmid pET_PAD-His by PCR using the primers EM05 and EM06. pET_ST-PAD-ST-His: The plasmid pET_PAD-ST-His was amplified by PCR using the primers EM07 and EM08 resulting in a linearized plasmid backbone which was then complemented to the full-length PAD with a DNA-fragment containing an additional N-terminal ST-GGGGS and matching overlaps (synthesized via “GeneArt Gene Synthesis” by ThermoFisher, Waltham, MA, USA) using Gibson assembly, as described above.

### 2.3. Gene Expression and Protein Production

Chemically competent *Escherichia coli* BL21 (DE3) were transformed with the corresponding expression vector. *E. coli* cells containing the expression vector were selected overnight at 37 °C on lysogeny broth (LB)/agar plates containing 100 µg/mL ampicillin. For expression, a single colony was transferred to a suitable volume of LB medium containing 100 µg/mL ampicillin and the culture was incubated overnight at 37 °C and 180 rpm in a shaking incubator. The overnight culture was then used to inoculate a 20 times larger volume of 25 °C warm ampicillin containing LB medium. This culture was then incubated for approximately 1.5–2 h up to an OD_600_ of 0.6–0.9 at 37 °C and 180 rpm. After reaching the appropriate cell density, the culture was cooled down to 25 °C for at least 15 min and subsequently induced with isopropyl β-D-1-thiogalactopyranoside (IPTG) to a final concentration of 0.5 mM. The induced culture was then incubated overnight at 25 °C and then harvested by centrifugation (10,000× *g*, 10 min, 4 °C). The cells were then resuspended in cold sodium phosphate buffer containing 10 mM imidazole (NPI 10; 50 mM NaH_2_PO_4_, 500 mM NaCl, 10 mM Imidazole, pH 8) and frozen at −80 °C. 

### 2.4. Protein Purification

The cell pellets were thawed at 25 °C in a water bath and then incubated with DNaseI and lysozyme for 30 min at 25 °C. Further cell disruption was then carried out using ultrasonification. The lysate was subsequently centrifuged for 1 h at 45,000 rcf and 4 °C. The pellet was discarded and the supernatant sterile-filtered through a 0.45 µm Durapore PVDF membrane with Steriflip^®^ Filter Units (Merck, Darmstadt, Germany). For protein purification, the cleared lysate was loaded on 2 × 5 mL His_60_ Ni Superflow Cartridge (Clontech, Palo Alto, CA, USA) mounted on an Äkta Pure liquid chromatography system (GE Healthcare, Freiburg, Germany). The purification was carried out with NPI 10 as running buffer and sodium phosphate buffer containing 500 mM imidazole (NPI 500;50 mM NaH_2_PO_4_, 500 mM NaCl, 500 mM Imidazole, pH 8) as elution buffer. After applying the lysate onto the column, the column was washed with 2% NPI 500 and the protein was eluted using a linear gradient (2% to 100% NPI 500). The column outflow was collected in 900 µL fractions and protein containing fractions (detected at 280 nm) were pooled. Subsequently, the buffer was exchanged to phosphate buffered saline (PBS) using Vivaspin 20, 5000 MWCO concentrators (Sartorius, Göttingen, Germany).

### 2.5. Sodium dodecyl sulfate–polyacrylamide gel electrophoresis (SDS-PAGE) Analysis

Samples were mixed with 4× SDS-PAGE loading buffer (200 mM Tris-Cl, pH 6.8, 400 mM DTT, 8% SDS, 0.4% bromophenol blue, 40% glycerol), boiled at 95 °C for 10 min and loaded onto a 1 mm thick SDS-PAGE gel containing 16% acrylamide. The gels were run at 100 V and stained with coomassie Brilliant Blue G-250. PageRuler (Plus) Prestained (Thermo Fisher Scientific) was used as molecular weight reference marker.

### 2.6. Determination of Decarboxylase Activity

Fifty µL of an enzyme solution in KP_i_ buffer (25 mM KP_i_, pH 6) were transferred in an ultraviolet (UV) transparent 96 well microtiter plate and 150 µL of *p*-coumaric acid to a final concentration of 1 mM in KP_i_ buffer were added. The consumption of *p*-coumaric acid was recorded at 294 nm using a Synergy MX microplate reader (BioTek, Winooski, VT, USA) over a period of 10 min at 25 °C. The activity was determined by calculating the slope in the linear range of the decrease of the absorption intensity (OD/min).

### 2.7. Hydrogel Prepration

Protein solutions of PAD-SC and PAD-ST/ST-PAD-ST were diluted in KP_i_ buffer to a final total protein concentration of 1 mM in 20 µL for analysis of ST/SC complex formation. For microrheology measurements a total volume of 200 µL was used and 0.2 mg/mL “dragon” green fluorescent polystyrene microspheres (200 nm diameter; Bangs Laboratories, Fishers, IN, US) were added. Polymerization was carried out for 1 h at 30 °C, 1000 rpm in a thermoshaker. Subsequently, the buffer was evaporated under constant centrifugation at 2200× *g* for overnight at 30 °C. 

### 2.8. Microrheology Measurements

Prior to multiple particle tracking (MPT)-analysis, the dried hydrogel samples were swollen by adding 25 µL KP_i_ buffer for 30 min under continuous shaking at 25 °C, 500 rpm. To perform MPT experiments, we used green fluorescent polystyrene microspheres of 200 nm diameter as tracer particles. Images of the beads were recorded onto a personal computer via a sCMOS camera Zyla X (Andor Technology, 50 f/s, Belfast, Northern Ireland) mounted on an inverted fluorescence microscope (Axio Observer D1, Zeiss, Kohen, Germany) equipped with a Fluar 100×, N.A. 1.3, oil-immersion lens. Movies of more than 100 fluctuating microspheres were analysed using the software Image Processing System (iPS, Visiometrics, Terrassa, Spain) and a self-written Matlab code, based on the widely used Crocker and Grier tracking algorithm [54]. 

To characterize sample heterogeneities, we examined the distribution of mean square displacements (MSDs), known as Van Hove correlation function [55] and calculated the non-Gaussian parameter *α* [56]:

(1) $$\alpha =\frac{\langle x^{4}(\tau )\rangle }{3\langle x^{2}{(\tau )\rangle }^{2}}-1$$

where *x* is the distance of particle center of mass along the *x* coordinate. This quantity is zero for a Gaussian distribution which is expected for a homogeneous and uniform sample, while higher *α* values reflect the presence of spatial heterogeneities.

### 2.9. High Performance Liquid Chromatography (HPLC) Analysis

All high performance liquid chromatography (HPLC) analyses were performed on an Agilent Technologies (Palo Alto, CA, USA) 1260 Bioinert Series with autosampler and diode array detector (DAD). *P*-hydroxystyrene and *p*-coumaric acid were detected and quantified by reverse phase HPLC using an Eclipse XDB C18 column (5 µm, Agilent) with a precolumn of the same material. The separation was realized at 10 °C with an isocratic mobile phase consisting of 60% acetonitrile and 40% ddH_2_O with 0.1% (v/v) trifluoroacetic acid. The flow rate was 1.5 mL/min. Absorption was detected simultaneously at 260, 275 and 370 nm and a suitable wavelength for calibration and evaluation was chosen for each substrate. For calibration, dilutions of *p*-hydroxystyrene and *p*-coumaric acid in the range of 0.5–5 mM were prepared. For measurements, 150 μL of each sample were diluted with 150 μL acetonitrile additionally containing 40 µM *p*-hydroxyazobenzene as internal standard and 250 mM HCl prior to application.

### 2.10. Microfluidic Setup and Analysis of the Hydrogels Under Continuous Flow

The microfluidic reactor was prepared as previously described [44]. The upper part containing the reaction channel was prepared by pouring of polydimethylsiloxane (PDMS) (Sylgard 184, Dow Corning, Midland, MI, USA) over molds to generate replicas. The channel architecture featured a straight channel which was 3 mm wide, 1 mm high and 54 mm long and a total volume of 150 µL. To serve as spacers for later fluidic connectors, cannulas (Sterican, B. Braun Melsungen AG, Melsungen, Germany) were integrated in the brass replica molds. The PDMS was cured at 60 °C for at least 3 h.

The microreactor was typically mounted inside an incubator (set to 30 °C), filled with 150 µL 1 mM protein solution (PAD-SC/ST-PAD-ST 2:1) and incubated for 60 min. The PDMS chips were then sealed with a polyolefin foil (HJ-BIOANALYTIK GmbH, Erkelenz, Germany). Low pressure syringe pumps (neMESYS 290N) equipped with 10 mL syringes were connected to a manual switching valve that was connected to the reactor for perfusion of reaction media at a flowrate of 10 µL/min. The syringes were filled with 10 mL substrate solution containing 5 mM of *p*-coumaric acid in KP_i_ buffer and 0.01% (v/v) sodium azide to avoid fouling. The reactor outflow was connected to the Compact Positioning System rotAXYS360 (CETONI, Korbussen, Germany) to allow for automatic fractioning into 96-well plates, previously loaded with 150 µL 250 mM HCl in acetonitrile to stop all enzymatic reactions and 40 µM *p*-hydroxyazobenzene used as standard for internal calibration. The flow was generated using a CETONI neMESYS Base module. Syringe pump and positioning system were controlled by the QmixElements-Software. The flow modules were connected via tubing (inlets: silicone Tygon tubing R3603, ID = 1.6 mm, Saint-Gobain, Cavaillon, France; outlets: conventional PTFE tubing, ID = 0.5 mm).

## 3. Results and Discussion

**Figure 2:**
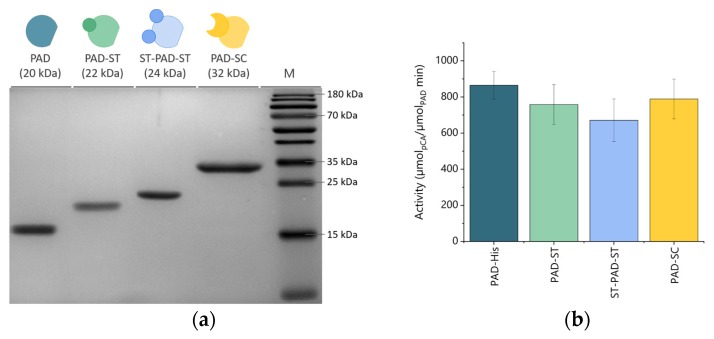
Variants of phenolic acid decarboxylase (PAD) from *Enterobacter* sp. containing either one or two SpyTag (ST) or one SpyCatcher (SC) were expressed in *Escherichia coli*, purified and tested for their activity towards *p*-coumaric acid. Note that all proteins carry a C-terminal 6× His-Tag. (**a**) Denaturing 16% SDS-PAGE analysis of the purified PAD constructs. The PAD variants used here are homodimers in their native form. Lane 1: PAD (20 kDa), Lane 2: PAD-ST (22 kDa); Lane 3: ST-PAD-ST (24 kDa); Lane 4: PAD-SC (32 kDa); Lane 5: PageRuler Prestained Protein Ladder (Thermo Scientific). (**b**) Enzymatic activity at 25 °C in µmol*_p_*_CA_/(µmol_PAD_ min) of the four differently tagged PAD variants against 1 mM *p*-coumaric acid (*p*CA) as substrate, determined by an absorbance based depletion assay.

In order to develop a PAD-based all-enzyme hydrogel, we initially generated expression plasmids for PAD variants that were genetically fused with polypeptide domains of SC or ST. Overexpression in *E. coli* and purification via an additional 6× His-Tag allowed to obtain the biocatalytically active recombinant proteins in near homogeneous purity, as judged from SDS-PAGE analysis (Figure 2a). This high purity allowed us to later produce materials with precisely defined properties. All variants expressed with very high expression yields ≥100 mg/L culture in a non-optimized process. To assess the catalytic performance of these PAD species, a first benchmark test was performed following the decrease of substrate by monitoring its absorption at 294 nm. The results in Figure 2b show the direct comparison of the activity of the various enzyme variants to convert the model substrate *p*-coumaric acid. It is clearly evident that all variants show comparable activities, however, the addition of one or two tags slightly decreased the activity as compared to the enzyme without an additional tag (dark blue bar, Figure 2b). 

**Figure 3:**
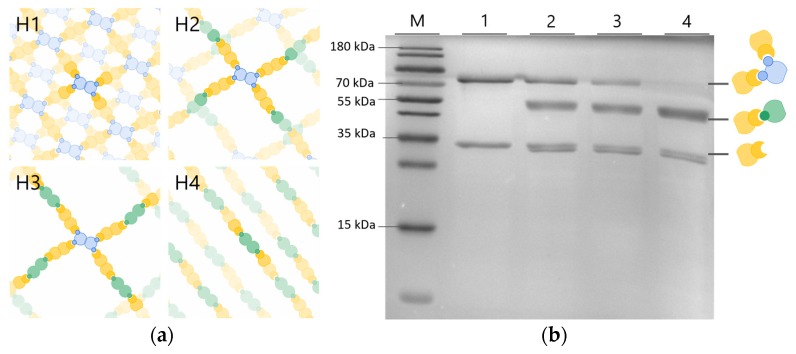
(**a**) Different types of PAD-Hydrogels (H1–H4) with different degrees of crosslinking can be prepared by using different stoichiometries of the SC/ST-tagged PAD variants. The various different hydrogels are depicted here as simplified and idealized 2D-representations with the individual building blocks approximately drawn to scale. The molar ratios of PAD-SC:PAD-ST:ST-PAD-ST were 2:0:1 (H1), 4:2:1 (H2), 6:4:1 (H3), 1:1:0 (H4). For each assembly, one repeating unit within the gel is highlighted. (**b**) Denaturing 16% SDS-PAGE analysis of the various types of hydrogels (H1–H4). The assembly reaction was carried out for 30 min at 30 °C and 1000 rpm and was quenched by the addition of 4× SDS-PAGE loading dye and incubation at 95 °C for 10 min.

Since the PAD enzyme naturally occurs as a homodimer we generated variants with one (PAD-ST) or two SpyTags (ST-PAD-ST) in order to study self-assembly properties with the complementary partner containing one SpyCatcher (PAD-SC) per monomeric subunit. This strategy should allow us to vary the degree of crosslinking and thus mesh size of the hydrogel by adjusting the stoichiometric molar ratios of the different enzyme variants, as illustrated in Figure 3a. Several types of PAD-hydrogels (H1–H4, Figure 3) were produced. For type H1 hydrogel, only PAD-SC and ST-PAD-ST were mixed in a 2:1 molar ratio, which should result in the highest possible degree of crosslinking. For type H2, prepared with a ratio of 4:2:1 of PAD-SC:PAD-ST:ST-PAD-ST, a lower degree of crosslinking was expected. Likewise, type H3, formed from a molar ratio of 6:4:1 of PAD-SC:PAD-ST:ST-PAD-ST, should display a further reduced degree of crosslinking. In type H4, where no double-tagged ST-PAD-ST was used and the PAD-SC:PAD-ST ratio was adjusted to 1:1, linear chains of crosslinked proteins should be formed without any branching points. 

To evaluate the ST/SC coupling in these systems, the formation of the covalent isopeptide bond was monitored by SDS-PAGE analysis (Figure 3b). Indeed, the expected dimeric and trimeric PAD conjugates were clearly formed. As expected, the ST-PAD-ST variant, which contains two crosslinking handles, formed trimeric conjugates with monomers of the PAD-SC, as indicated by the dominant band of approximately 87 kDa in lane H1. This result also proves the accessibility of both the N- as well as C-terminal SpyTag peptide in the ST-PAD-ST construct. In contrast, no higher conjugates were observed when single-tagged PAD-ST was used (lane H4). 

**Figure 4:**
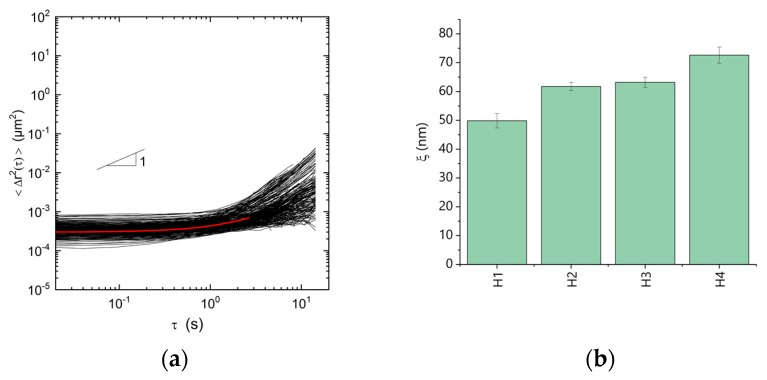
Multiple particle tracking (MPT) microrheology analysis of local viscoelastic properties of different types of PAD hydrogels (H1–H4, specified in Figure 3). (**a**) Mean square displacements (MSDs) (black curves) of individual fluorescent marker beads of diameter 200 nm dispersed in the hydrogel H1. The red curve is the ensemble-average MSD. (**b**) Comparison between mesh size values *ξ* as deduced from MPT experiments for the different hydrogels. All experiments were carried out in duplicates. Note that the experimentally determined mesh correlate with the fraction of crosslinking proposed for hydrogel samples H1–H4.

Microrheological experiments were carried out to confirm the polymeric nature of the hydrogels and to quantitatively determine the degree of crosslinking in the various H1–H4 materials in order to identify the most stable gel for the subsequent flow experiments (Figure 4). All hydrogels were obtained as monolithic, elastic materials with rheological properties comparable to hydrogels prepared from different enzymes, as reported previously by our group [44,46]. Figure 4a shows the variation of mean square-displacements (MSDs) as a function of lag time for polystyrene particles with a diameter of 200 nm dispersed in the hydrogel H1. All MSDs are almost independent of time (slope ≈ 0) for times *τ* < 1 s with a narrow distribution of absolute values characterized by a non-Gaussian parameter α ≈ 1. This result is consistent with an elastic trapping of tracer particles in a homogeneous gel-like network. Similar results were obtained for all hydrogels investigated. The calculation of the elastic shear modulus *G*_0_ using the relation *G*_0_ = 2*k*_B_*T*/3*πα*Δ*r*^2^ where *k_B_* is the Boltzmann constant, *T* the temperature, *α* the tracer particle radius and Δ*r*^2^ the time-independent average MSD exhibits a gradual decrease of *G*_0_ from 33 ± 5 via 17 ± 2 and 16 ± 1 to 11 ± 1 Pa for H1, H2, H3 and H4, respectively. The higher elasticity obtained for H1 is presumably due to the fact that H1 contains the highest amount of divalent ST-PAD-ST whereas H4 is built solely from fusion proteins with only two binding valences, which suggests in the latter case the occurrence of much less crosslinking events. Finally, we directly determined the mesh size *ξ* of the network according to the classical theory of rubber elasticity with $G_{0}=\frac{k_{B}T}{\xi^{3}}$ and we found a significant *ξ* increase from 50 ± 3 nm for H1 to 73 ± 3 nm for H4 (Figure 4b), which corresponds with a decrease of the crosslinking proportion. Hence, the significantly larger elasticity and smaller mesh size of H1 reflects the greater connectivity that originates from the higher amounts of ST-PAD-ST providing four valences as branching points. Hydrogel H1, as the most tightly connected material, was selected as the formulation for the subsequent experiments in the flow reactor. Altogether, this study also illustrates that the rheological properties of the novel all-enzyme hydrogels can be rationally modulated by adjusting the stoichiometric crosslinker ratio.

**Figure 5:**
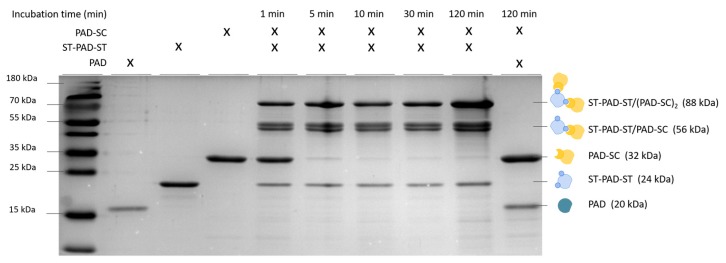
Kinetic analysis of the ST/SC-conjugation of ST-PAD-ST and PAD-SC analyzed by coomassie stained 16% SDS-PAGE. Stoichiometric amounts (30 pmol) of both enzymes were incubated for up to 2 h at 30 °C. The reaction was stopped by the addition of 4× SDS-PAGE loading buffer and heat inactivation, which leads to denaturation of non-covalent protein interactions. As expected, the covalent bond between ST-PAD-ST and PAD-SC is rapidly formed upon mixing, leading to the formation of dimeric and trimeric conjugates with sizes of 55 kDa and 87 kDa, respectively. Note that PAD-SC (32 kDa) does not react with PAD (20 kDa) even after a prolonged incubation of 120 min (lane 10).

To shed light on the assembly kinetics, the ST/SC-mediated gelation of PAD-SC with the double-tagged ST-PAD-ST was further investigated by SDS-PAGE analysis (Figure 5). The results indicated that the coupling reaction is almost completed within the first few minutes after mixing. In contrast, no polymerization occurred even after prolonged incubation times in control reactions with variants lacking complementary binding sites (lane 10 in Figure 5). Hence, these results clearly confirm and emphasize the excellent coupling capabilities of the PAD variants, thereby emphasizing the specificity and high reaction rates of the ST/SC system [47]. 

**Figure 6:**
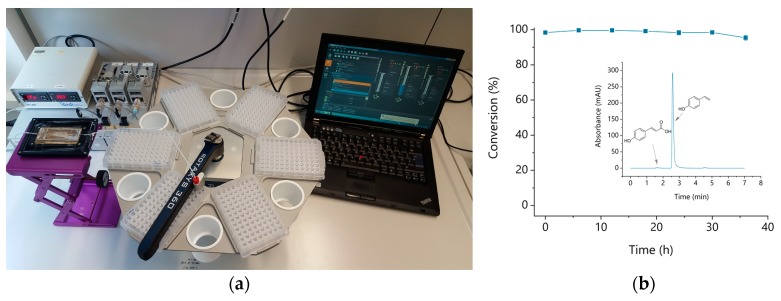
Continuous flow, biocatalytic production of p-hydroxystyrene using PAD hydrogel-loaded microreactors. (**a**) Microfluidic set-up used in this study. (**b**) Time dependent synthesis of *p*-hydroxystyrene, determined from the outflow of the enzyme-loaded reactor that was perfused with 5 mM substrate at a flowrate of 10 µL/min at 25 °C. The PDMS reactor had a total volume of 150 µL. The shown data was determined by reverse phase HPLC (inset) from two separate experiments.

We then used the polymerization of the PAD variants to produce biocatalytic hydrogels that were mounted into miniaturized flow reactors in order to facilitate the continuous synthesis of *p*-hydroxystyrene (Figure 6). To this end, syringes containing a 5 mM solution of *p*-coumaric acid were connected to PDMS microreactors loaded with hydrogel H1 and the substrate solution was perfused at a flow rate of 10 µL·min^−1^ with automated sampling of the outflow (Figure 6a). The microreactor architecture featured a reactor volume of 150 µL with dimensions of 3 × 1 × 54 mm. The PAD-based all-enzyme hydrogel showed a stable and almost complete conversion for time periods of >10 h (Figure 6b) with only traces of the substrate remaining in the product samples, as determined by reverse phase HPLC (inset in Figure 6b). The high and constant conversion rates even after longer runtimes indicate that no significant enzyme leaching takes place, which would otherwise lead to a constant decline in turnover. Without further optimization, a typical space time yield (STY) of 57.7 g·(d·L)^−1^ with conversions ≥98% was obtained. Hence, this initial proof-of-concept study clearly indicated that the novel PAD-based all-enzyme hydrogels hold a great potential for further optimization and application in biocatalysis.

## 4. Conclusions

In conclusion, we herein describe a novel material, a self-assembling all-enzyme decarboxylase hydrogel, that was used for biocatalytic flow synthesis of *p*-hydroxystyrene, which in turn can be used for the synthesis of fine chemicals and pharmaceuticals. We here demonstrate for the first time, that a phenolic acid decarboxylase from *Enterobacter* sp. can be genetically fused with either ST or SC polypeptide domains without compromising the enzyme’s catalytic performance. We also demonstrate that the biocatalytic hydrogels can be adjusted in terms of their rheological properties and mesh size by simply varying the stoichiometric molar ratio of the interconnecting fusion partners. Further in-depth studies of the modular composition and the resulting changes in polymer properties will be used to optimize the biocatalytic performance and compare it with other immobilization methods. The proof-of-concept results presented here already clearly show that the PAD-based hydrogels are excellently suited for applications in flow biocatalysis. This enabled us to generate a conversion ≥98% in a straight channel reactor with a volume of 150 µL and a space-time yield of 57.7 g·(d·L)^−1^. Therefore, our study does not only expand the scope of all-enzyme hydrogels by adding a new enzyme with high application relevance to the toolbox. The present work also suggests that our approach holds great potential for the establishment of advanced microfluidic manufacturing processes that include numbering up of chips to improve productivity [46] or the implementation of biocatalytic and chemoenzymatic cascades in machine-assisted compartmentalized production processes [32].

## Figures and Tables

**Table 1:** Primers used in this study.

Primer	DNA-Sequence
EM01	GGTCATCATCACCATCATCATTAAGATCC
EM02	GCTACCACCACCACCTTTCAGATTATC
EM03	CCGGATAATCTGAAAGGTGGTGGTGGTAGCGTTGATACCCTGAGCGGTCTGAG
EM04	CGGATCTTAATGATGATGGTGATGATGACCAATATGTGCATCACCTTTGGTTGCTTTACC
EM05	TGGTTGATGCATATAAACCGACCAAAGGTCATCATCACCATCATCATTAAGATCC
EM06	CGGTTTATATGCATCAACCATAACAATATGTGCGCTACCACCACCACCTTTCAGATTATC
EM07	CACAATTCCCCTATAGTGAGTCGTATTAATTTC
EM08	GTGAAAGCATCTATAAAATCAGCTGGACC
